# The Influence of Vegan, Vegetarian, and Omnivorous Diets on Protein Metabolism: A Role for the Gut–Muscle Axis?

**DOI:** 10.3390/nu17071142

**Published:** 2025-03-26

**Authors:** Waed Al-Refai, Stephen Keenan, Donny M. Camera, Matthew B. Cooke

**Affiliations:** 1 Department of Health and Biostatistics, School of Health Sciences, Swinburne University, Melbourne, VIC 3122, Australia; walrefai@swin.edu.au; 2 Department Sport, Exercise and Nutrition Sciences, School of Allied Health, Human Services and Sport, La Trobe University, Bundoora, VIC 3086, Australia; s.keenan@latrobe.edu.au

**Keywords:** muscle protein synthesis, vegan, omnivorous, gut microbiome, exercise, skeletal muscle

## Abstract

There has been a growing interest globally in vegan and vegetarian diets over the last decade for a combination of health, ethical, environmental, spiritual, and social reasons. In line with this popularity, research examining the role of plant-based food sources, including vegan and vegetarian diets, in supporting skeletal muscle remodeling and anabolism in humans has also received considerable attention. The emergence of the microbiota-gut–muscle axis, a bidirectional pathway where the gut microbiota impacts skeletal muscle and vice versa, has been suggested as a potential mediator of food and nutrition’s influence on the mechanistic processes that regulate muscle mass and function. Considering inherent nutritional differences between vegan, vegetarian, and omnivorous diets related to the fiber and macronutrient content, presence of anti-nutritional factors, and diverse food and supplemental sources for obtaining protein, it stands to reason that the regulation of the microbiota–gut–muscle axis via diet-induced changes in gut microbiota composition and function may be dissimilar. However, whether this translates into differential effects on the skeletal muscle is unclear. This review article aims to provide a contemporary perspective for how variations in gut microbiota linked to vegan, vegetarian, and omnivorous diets may be a potential mechanism for influencing protein metabolism in skeletal muscle mass via a purported microbiota-gut–muscle axis.

## 1. Introduction

Over the past few years, there has been a growing interest in vegan and vegetarian diets in many Western countries, including the United Kingdom, the United States, and Australia [1]. This increase can be attributed to several factors, namely, health, ethical, environmental, spiritual, and social. Vegan diets are not to be mistaken for a vegetarian diet, which allows a small-to-moderate amount of animal products to be consumed (i.e., a flexitarian diet, lacto-ovo-vegetarian, etc.). More recently, the term “plant-based” diet has received a lot of interest coinciding with the publication of the EAT–Lancet report in 2019 [2]. Similar to other initiatives where recommended dietary guidelines are presented visually on a plate schematic, the report recommended a “planetary health” plate that consists of approximately half a plate of vegetables and fruits, with the remainder made up of whole grains, plant-based protein sources, unsaturated plant oils, and smaller amounts of dairy, animal proteins, starchy vegetables, and added sugars [2,3]. The term plant-based diet is often used interchangeably with vegan diets, implying that both are characterized by the avoidance of all flesh foods and animal-derived ingredients [4]. On the contrary, others explicitly note that the term plant-based diet does not mean vegan but rather prioritizes foods that come from plants [5]. Regardless, accumulating evidence suggests that incorporating more plant-based options into a habitual diet can have numerous health benefits and lower risk of cardiovascular disease and cancer [2,6,7].

The popularity of vegan and vegetarian diets amongst athletic populations, especially endurance athletes, continues to grow, with previous studies observing that 10% of marathon runners [8] and 13% of ultramarathon runners [9] follow vegan or vegetarian diets. While more athletes appear to be adopting vegan and vegetarian dietary lifestyles, such diets can lead to inadequacies in specific macro- and/ or micronutrients (i.e., protein, n-3 fatty acids, Vitamin B12, iron, etc.) compared to an omnivorous diet and, as such, may negatively impact performance and training adaptations [10]. Conversely, others claim that any dietary deficiencies are likely due to poorly constructed diet plans rather than vegan and vegetarian diets [1,11]. In fact, well-balanced vegan and vegetarian diets can provide adequate amounts of these macro- and micronutrients and, in some situations, even greater amounts of carbohydrate and antioxidant-rich foods compared to omnivorous diets, thus assisting in training and recovery [12].

The role of vegan and vegetarian diets in supporting resistance training-induced skeletal muscle remodeling and anabolism in humans has also received recent attention. Studies examining isolated plant proteins (soy, pea, wheat, and corn) have demonstrated increased muscle protein synthesis responses following intake, both with and without exercise [13,14,15]. While most research in this area has focused on isolated protein supplements, emerging evidence suggests that whole-food plant meals can support anabolic muscle adaptations, especially when adequate vegan/plant protein intake is provided. The interplay between diet and exercise is complex, with many exogenous factors such as exercise (sets, repetitions, volume, and frequency) and diet (protein type and amount and energy intake), as well as endogenous factors, including genomic, epigenetic, transcriptomic, and proteomic regulation, all influencing the magnitude of muscle hypertrophy and stimulation of muscle protein synthesis (MPS) with exercise. The gut microbiome has recently been identified as another important factor, with the emergence of the microbiota–gut–muscle axis. Evidence from both animal and, to a lesser degree, human models of sarcopenia suggest a bidirectional relationship between microbiome and skeletal muscle physiology exists [16,17]. Modulation of the immune system, reductions in oxidative stress and inflammation, and/or the production of short-chain fatty acids (SCFA) (especially butyrate) and pro-anabolic mediators (i.e., increased availability of systemic amino acids and uptake) have all been suggested as possible mechanisms of action by which the microbiota directly and/or indirectly influences muscle physiology [18]. Given vegan and vegetarian diets can be higher in fiber, polyphenols, and other “healthy” microbiome-promoting ingredients compared to omnivorous diets, it could be hypothesized that vegan and vegetarian diets are superior for enhancing the microbiota–gut–muscle axis [19,20]. However, whether this translates into greater anabolic adaptations in muscles with exercise is unclear. Conversely, omnivorous diets, which are higher in animal proteins and thus essential amino acid (EAA) profiles that are more optimally suitable for muscle uptake and assimilation into muscle tissue, could be superior in promoting muscle adaptations to resistance exercise, independent of significantly modulating the microbiota–gut–muscle axis.

The primary objective of this narrative review is to, therefore, assess variations in gut microbiota linked to vegan, vegetarian, and omnivorous diets as potential mechanisms for influencing acute MPS responses in skeletal muscle mass via the purported microbiota–gut–muscle axis. We first compare the effects of vegan, vegetarian, and omnivorous diets on various aspects of the gut microbiome, including microbial diversity, composition, and metabolites, along with identification of specific bacteria or bacterial “signatures” (multiple bacteria genera) and link these to mechanisms of action of the microbiota–gut–muscle axis. Subsequently, we will discuss the direct effects of plant-based protein sources, including vegan, vegetarian, and omnivorous diets, on MPS responses to put forward putative evidence that may link the direct physiological action of distinct gut microbiota phenotypes associated with plant- and meat-based dietary interventions with the stimulation of MPS in adults.

## 2. Effects of Vegan, Vegetarian, and Omnivorous Diets on Gut Microbiota Diversity, Composition, and Associated Bacteria-Derived Metabolites

### 2.1. Modulation of the Microbiome with Diet

It is well-known that the organisms within a microbiome consume the same food as the host, so it is unsurprising that diet affects the composition of microbes present in the human intestinal tract. The long-term dietary habits of an individual, such as the consumption of protein and animal fat versus carbohydrates, are key factors in determining their core microbial composition. However, both rapid and drastic changes in dietary habits can cause detectable shifts in the subsets of microorganisms within 24 h, although these changes are not considered permanent and require continued consumption of the altered diet to be maintained [21]. Over longer periods, evidence suggests that the microbiome co-evolves with the host’s diet to maximize nutrient utilization [22,23].

The impact of vegan and vegetarian diets on gut microbiota, microbial fermentation products, and subsequent host physiology is a topic of debate with no clear consensus. To date, the majority of information comes from cross-sectional studies, with few well-designed intervention studies published. It is currently unclear whether the health benefits of vegan and vegetarian diets are solely due to their nutritional composition or also partly due to impacts on the gut microbiome. Notwithstanding, the following section summarizes recent findings from both observational and intervention studies in humans, focusing on differences in microbiota composition and/or associated bacteria-derived metabolites between vegan, vegetarian, and omnivorous diets. The characteristics of cross-sectional and interventional studies can be found in Table 1 and Table 2, respectively.

### 2.2. Cross-Sectional Studies Comparing Vegan and Omnivorous Diets

The earliest study to investigate the relationship between diet and gut microbiota was in the early 1970s, with diets high in meat (“mixed Western diet”) compared to a not-so-well-defined “non-meat diet” [24]. Using a very small number of participants, eight volunteers switched their usual mixed Western diet to a non-meat diet for four weeks. Though not a cross-sectional study by definition, this study found that those who consumed meat displayed an anaerobic microbiota profile enriched in *Bacteroides*, *Bifidobacterium*, *Peptococcus*, and *Lactobacillus* compared to non-meat eaters. Approximately 37 years after this study by Reddy et al. [24], Zimmer and colleagues [25] set out to distinguish differences in the fecal microbiota profiles of adult vegans, vegetarians, and omnivores (control) within a German cohort that were age and sex-matched. Self-proclaimed adherence to both vegan and vegetarian diets was short and only 4 weeks prior to the study. Fecal pH was significantly lower in vegans compared to omnivores, which was strongly correlated with reduced counts of *E. coli* and *Enterobacteriacea* species that are not tolerant of an acidic environment [25]. Low fecal pH is also likely a reflection of higher fiber intake within vegans, as shown in more recent studies [32]; however, fiber and dietary intake were not measured in the study by Zimmer and colleagues. Longer term adherence (minimum one year) within a Slovenian adult population showed higher ratios (percentage of group-specific DNA in all bacterial DNA) of *Bacteroides–Prevotella*, *Bacteroides thetaiotaomicron*, *Clostridium clostridioforme*, and *Faecalibacterium prausnitzii* in vegetarians and/or vegans compared to omnivores [27]. Additionally, both vegan and vegetarian participants displayed a lower ratio of *Clostridium* cluster XIVa when compared to omnivores, a result similarly noted in another study in vegetarian women from southern India [26]. *Faecalibacterium prausnitzii* has been identified as a key bacterial strain linked to skeletal muscle mass, given it is a butyrate producer and displays anti-inflammatory properties [42].

A series of independent studies compared gut microbiota profiles between vegan, vegetarian, and omnivorous populations in Italy. Lossaso and colleagues observed higher counts of the phylum Bacteroidota (synonym Bacteroidetes) in vegans and vegetarians compared to omnivores (all of which had been strictly following their respective diets for more than 12 months) [31]. Bacteroidota phylum are typically commensal bacteria and proficient in polysaccharide fermentation that leads to the production of SCFAs and other important molecules. However, at the genus level of this bacteria, contrary findings were observed in the aforementioned studies by Reddy [24] and Zimmer [25], as well as more recent studies [28,32], with a lower abundance of *Bacteroides* in vegans compared to omnivores. Furthermore, Tarallo and colleagues observed lower counts of *Bacteroides* but at the species level, with a lower abundance of *Bacteroides dorei* (currently *Phocaeicola dorei*) in vegans compared to omnivores [35]. Despite higher abundance levels at the phylum level, at the lower levels (i.e., species), reduced abundance could indicate a lack of diversity. This could reflect a diet low in protein and animal fat and high in fiber, as comparable patterns in *Bacteroides* abundance have been observed across both the short-term (4 weeks) [24,25] and long-term (1+ year) [28,31,32,35].

Geographic location is an important consideration when studying the impact of diet on the microbiome and resistome composition, as even different locations within the same country (i.e., north versus south regions) can have a significant impact. Within a Dutch cohort, differences in the relative abundance of lactic acid bacteria were observed when comparing microbiomes between diets, with omnivores and pescatarians displaying higher levels of *Streptococcus thermophilus* and *Lactococcus lactis* (pescatarian only) when compared to vegans [34]. Given the relatively high consumption of dairy products in the Netherlands [43], it is not surprising to see omnivores and pescatarians (who include dairy products in their diet) demonstrate higher levels compared to vegans (excluded from such diet). Prochazkova and colleagues used a multi-omics approach to investigate differences between self-reported (past 3 years) vegans and omnivores within central Europe [33]. Ten genera that belong to the core microbiome were different between groups, with enrichment of *Lachnospira*, *Lachnospiraceae* NK4A136 group, and *Ruminiclostridium* and depletion of *Alistipes*, *Bifidobacterium*, *Blautia*, *Fusicatenibacter*, *Dorea*, *Anaerostipes*, and Ruminococcaceae uncultured groups in vegans compared with omnivores. There were also several differences in the fecal metabolome between vegans and omnivores. Specifically, vegans displayed lower levels of the amino acid fermentation products p-cresol, scatole, indole, and methional, but higher levels of polysaccharide fermentation produced SCFAs and medium-chain fatty acids (MCFAs) and their derivatives. Reported levels of SCFA concentrations in populations following a vegan diet over the long term have been indifferent. While a study by De Filippis and colleagues demonstrated significant associations between the consumption of vegetable-based diets and increased levels of fecal SCFAs [29], a study by Trefflich and colleagues failed to show any difference in fecal SCFA and branch-chain fatty acid (BCFA) levels between vegans and omnivores [32]. This is also in line with another cross-sectional study in a healthy Western vegan population who followed their diet for at least six months [30]. The authors suggested that the microbiota structure of the Western population is “restrictive” and SCFA production does not increase linearly with fiber availability, which contrasts with an agrarian society characterized by a more “permissive” microbiota structure [30].

Most recently, findings from a 24-month observational study in a German population showed no significant difference in the richness (alpha diversity) of the gut microbiome across vegan, vegetarian, and omnivorous diets when cross-sectionally analyzed using an ANOVA statistical approach [36]. While this is in line with previous studies [29,30], it is contradictory to others that have reported greater richness in vegetarians compared to omnivores [31], as well as greater alpha-diversity in non-vegans compared to vegans based on different measures of diversity [33]. Another notable finding from the German population study was that vegan and vegetarian dietary patterns differed significantly in their composition (beta diversity) when compared to omnivores. Omnivores displayed the highest abundance of the *Ruminococcus torques* group, *Eubacterium ruminantium* group, Ruminococcaceae, *Lachnospiraceae*_2, *Lactobacillus*, and *Senegalimassilia*, while these were lowest in vegans [36]. Others have also reported that *Ruminococcus* and the family of Ruminococcaceae were positively associated with an omnivorous diet [29]. *Ruminococcus* plays a role in converting animal-derived choline and carnitine, primarily sourced from eggs, beef, pork, and fish, into trimethylamine (TMA). The liver oxidizes TMA, releasing it as trimethylamine oxide (TMAO), a compound that has been associated with cardiovascular disease [44]. This contributes to the elevated urinary TMAO levels observed among omnivores [29,45]. Finally, in one of the largest cross-sectional studies, Fackelmann and colleagues [37] analyzed 21,561 individuals spanning five independent, multinational, human cohorts (including previously mentioned Italian cohorts) to map how differences in diet patterns (omnivore, vegetarian, and vegan) are reflected in gut microbiomes. Several species were higher in omnivore microbiomes and were linked to meat consumption (i.e., *Alistipes putredinis*) and inflammation (i.e., *Ruminococcus torques*), as previously observed [29,36]. In contrast, several species were over-represented in vegan microbiomes that are both known butyrate producers (*Lachnospiraceae*, *Butyricicoccus* sp., and *Roseburia hominis*) and highly specialized for fiber degradation (*Lachnospiraceae*) [37].

### 2.3. Intervention Studies Comparing Gut Microbiome Responses to Vegan and Omnivorous Diets

In contrast to observational studies, significantly fewer dietary intervention studies have explored and compared the short- or long-term impacts of vegan, vegetarian, and omnivorous diets on microbiota composition and/or function. The earliest studies to include a vegan diet as one of their experimental diet conditions was by Van Faassen and colleagues in 1987 [38]. In this study, the authors used a cross-over design to compare a vegan diet, lacto-ovo-vegetarian diet, and mixed Western diet for 20 days, each under very controlled conditions. Following the vegan diet, participants demonstrated significantly lower levels of fecal *Lactobacilli* and *Enterococci*, along with lower concentrations of bile acids, coprostanol, and coprostanol plus cholesterol compared to the mixed Western diet. While no details of the washout period were provided (limiting the ability to determine whether there were any carryover effects between interventions), it has been suggested that 2–3 weeks is enough time to return gut microbiota to pre-intervention states after short-term dietary alterations [21]. David and colleagues examined the short-term consumption (5 days) of diets composed entirely of animal or plant products on microbial community structure, short-chain fatty acid production, and microbial gene expression [39]. Although no significant differences in alpha diversity were detected in either diet, beta diversity was increased (within 24 h) following the animal-based diet. Further, the animal-based diet appeared to induce a more pronounced effect on the relative abundance of clustered species-level bacterial phylotypes (22 clusters vs. 3 clusters), specifically in bile-resistant taxa (*Bilophila wadsworthia*, Cluster 28; *Alistipes putredinis*, Cluster 26; and a *Bacteroides* sp., Cluster 29). The animal-based diet also led to a decrease in Firmicutes that break down plant polysaccharides (such as *Roseburia*, *Eubacterium rectale*, and *Ruminococcus bromii*). These microbial changes mirrored the differences seen in other studies [35,46] and suggest microbes find a compromise between carbohydrate and protein fermentation [39]. In contrast, Zhang and co-workers observed no effect on gut enterotype or gut microbiota alpha diversity by changing to a lacto-ovo-vegetarian diet (previously omnivorous) for 3 months [40]. However, beta diversity at the genus and species level did decrease. Such differences are likely due to the strictness of the diets, with the study by David and colleagues [39] only comparing plant (grains, fruits, and vegetables) or animal products (meats, eggs, and cheese), whereas the vegetarian diet in the work by Zhang and colleagues [40] was only devoid of meat, and the omnivores consumed both animal and plant products.

Finally, Kohnert and colleagues [41] examined 4 weeks of strict vegan and meat-rich diets in healthy adults. Both alpha and beta diversity were not significantly different between diets. Similarly, changes in gut microbiota composition were minimal after the intervention. The observed changes in amplicon sequence variants (ASVs) were attributed to a few participants, predominantly in ASVs occurring in less than 40% of samples. These were *Faecalibacterium* (unspecified species) and *Roseburia faecis*, which were depleted in the vegan diet, and *Blautia* (unspecified species) as well as *Faecalibacterium* (another unspecified species), which were enriched in the meat-rich diet. In contrast, a study by David and colleagues [39] found higher levels of *Roseburia* in vegetarians due to their higher fiber intake. However, factors beyond overall dietary intake patterns, such as specific food choices, can influence the abundance of butyrate-producing *Roseburia*, with wholegrain foods reported to enrich species.

### 2.4. Limitations of Current Evidence Comparing Gut Microbiome Responses Between Vegan and Omnivorous Diets

Contextualizing the findings from the above studies requires consideration of factors that may impact the gut microbiome and changes in the gut microbiome in response to dietary challenges. These include, but are likely not limited to, geographical location and environment (which impact baseline and long-term dietary patterns and subsequent microbiome profiles and responses to changes in diet) and individual differences, such as specific food choices. Furthermore, analytical methods and variability in study design also make it difficult to draw firm conclusions based on the current evidence base. A large proportion of the studies were based in Europe; however, even across regions of Europe, baseline dietary patterns varied considerably. The European country most represented was Italy [28,31,35], while others were conducted in Germany, the Netherlands, and Czechia [25,33,41]. Although on the same continent, typical dietary patterns in Italy are characterized by an increased intake of plant foods (except potatoes) and lower consumption of animal and processed foods, whereas this is the opposite in Germany and the Netherlands [47]. Similarly, dietary patterns in the US generally include even higher intakes of animal foods (in particular red and processed meat) [48], alongside low intake of plant foods, aside from (non-wholegrain) grains [49]. This is important given the possibility that certain “keystone” species may be required for the divergence in gut microbiota in response to increased dietary consumption of plant-based foods and that the presence of these species may vary depending on geographical location [30]. Although we could discern no clear pattern from the studies included, this remains an important consideration when interpreting the results of previous research.

While geographic location may exert a large influence over the dietary patterns of populations, even within those dietary patterns, specific dietary choices may also heavily influence the gut microbiome. As noted, significant consumption of dairy products in a Dutch population likely led to a greater relative abundance of lactic acid bacteria, while red meat is a strong driver of omnivorous microbiome signatures [37]. Although each of these food types might be consumed in an omnivorous diet and/or vegetarian diet (excluding meat), the influence of the relative proportions of these food types may not be obvious if this intake data is not captured. Similarly, given vegan microbiome signatures may be related to soil microbes [37], it is also likely that this may be influenced by food choices (e.g., organic versus non-organic, local versus non-local). This adds further complexity when interpreting the effects of vegan versus omnivorous diets on the gut microbiome.

The lack of consistency in analytic methods across studies also makes the interpretation of results difficult. For example, traditional 16S rRNA gene sequencing (a common PCR-based method) allows researchers to identify and quantify bacterial taxa at the genus level but offers limited insight into functional capabilities [50]. In contrast, shotgun metagenomic sequencing may provide greater resolution, including details on which genes the microbes carry and additional information on metabolic pathways associated with different community members [51]. Other methods focus on metabolite profiling, for example, measuring short-chain fatty acid production or amino acid byproducts, which provides important information on microbial activity and functionality, without necessarily identifying the organisms responsible. Multi-omics approaches often combine metagenomics, transcriptomics, proteomics, and metabolomics, giving a more comprehensive picture of the microbiome’s functional output [52]. While findings from different methods can be integrated to build a more comprehensive picture, it is crucial to interpret these results within the context of the limitations inherent to each analytical technique.

## 3. Effects of Vegan, Vegetarian, and Omnivorous Diets on Muscle Protein Synthesis Responses

The following section discusses studies that have investigated the effects of isolated plant-based (i.e., non-animal-derived) protein supplements, along with whole-food vegan meals and diets, on MPS responses, with comparisons to animal-based proteins/diets made where applicable. For the sake of simplicity, in the next section, non-animal sources are limited to protein sources of either plant or fungi origins, while omnivorous sources are based on food sources of animal origin, excluding alternative sources, such as insects, worms, etc. The characteristics of these studies can be found in Table 3.

### 3.1. Effects of Supplement Vegan and Omnivorous Protein Sources on Muscle Protein Synthesis Responses

The majority of research in this growing area has focused on the use of isolated protein supplement sources. Early work incorporated soy protein with one study in males aged in their early 20s, showing a greater MPS response with skimmed milk compared to soy protein (both containing ~18 g protein) after a single session of resistance exercise [53]. Another study by Tang and colleagues compared MPS responses between equal amounts of whey, casein, and soy protein (~20 g, with each providing ~10 g of EAAs) during recovery from resistance exercise in healthy young males [15]. Interestingly, although whey protein induced the greatest rise in MPS, the ingestion of soy protein induced a greater increase in MPS compared to the animal-derived casein protein. Another notable finding from this study was that the ingestion of whey and soy protein, both of which are acid soluble, which is important for their digestion in the acidic environment of the stomach, resulted in a more rapid appearance of leucine in the blood than with the ingestion of casein protein. In older adults aged in their early 70s, whey protein isolate in 20 g and 40 g amounts similarly induced significantly higher MPS responses compared to the same respective amounts of soy protein isolate in both a rested state and after a single bout of resistance exercise [54]. However, not all studies have reported lower MPS responses with soy protein compared to animal-based protein sources. A study in younger adults aged in their early 20s observed no differences in MPS responses when 45 g of carbohydrate was co-ingested with either 20 g of protein from whey, soy, or a leucine-enriched soy beverage following a single session of combined resistance and endurance exercise [67]. As this study was conducted in younger adults, it is possible that differences in soy and animal-based protein beverages may only be apparent in older adults as a result of “anabolic resistance” to generate similar MPS responses to equal amounts of protein compared to younger adults [55,74].

More recently, research led by the van Loon group has focused on the effects of other plant-based protein sources for stimulating MPS responses, such as potato, wheat, corn, and pea. Potato protein contains a relatively high level of EAAs (in particular, leucine) and, therefore, represents a promising type of plant-based protein to enhance MPS responses. In support, the ingestion of 30 g of either potato protein or milk protein increased MPS responses following a single bout of resistance exercise, with no differences in the magnitude of response between the potato- or milk-protein groups [57]. This amount of potato protein provided similar amounts of EAAs (~10.5 g), leucine (2.6 g), lysine (~2 g), and methionine (0.6 g) as the equivalent amount of milk protein, showing the intake of potato protein post-exercise can be an efficacious alternative to animal-based protein beverages. Thirty-gram amounts of either pea, wheat, or corn protein were similarly shown to induce comparable rates of MPS as 30 g of milk protein [14], as well as a blend of milk and wheat protein [13] or a blend of milk and corn protein [62] in healthy young males. Additionally, comparable increases in the post-exercise rates of myofibrillar protein synthesis were observed in young healthy adults between a 0.33 g/kg dose of fava bean protein and an EAA-free control beverage [59]. Interestingly, in this study, there were no increases in resting (i.e., non-exercise stimulated) rates of MPS with either beverage; thus, it is possible that the magnitude of post-exercise increase in MPS could have been significantly higher in the control group compared to the fava bean group if the control beverage contained adequate amounts of EAAs. The ingestion of different plant-based protein blends on rates of MPS has also been investigated. Pinckaers and colleagues showed that consuming 30 g of a plant blend protein (15 g wheat + 7.5 g corn + 7.5 g pea protein) in 24 healthy, young, recreationally active males induced comparable rates of MPS to an equivalent amount of milk protein despite an attenuated postprandial rise in circulating plasma essential amino acid concentrations (i.e., isoleucine, threonine, and valine) [60]. In contrast, higher rates of MPS were observed following the ingestion of 25 g whey protein compared to the same amount of a plant-based protein blend composed of 88% pea protein (contains a moderate amount of leucine and lysine but low amounts of methionine) and 12% canola protein (contains higher amounts of methionine) [71]. Notably, in this work, when the plant-based blend was enriched with an additional 1.5 g of leucine, there were no differences in MPS responses. While these findings collectively highlight the importance of an adequate EAA profile in a plant-based protein supplement/beverage for stimulating MPS responses, a caveat to the discussed studies is that they were all undertaken in young and healthy adults.

Older adults have been shown to present with a blunted postprandial MPS response to amino acid/protein intake, coined “anabolic resistance”, compared to younger adults, with the bulk of this work incorporating animal-based protein sources [74,75,76,77]. Thus, to address the capacity for anabolic resistance with plant-based/vegan protein sources, Gorissen and co-workers compared MPS responses to different amounts of wheat hydrolysate, casein, and whey protein in older adults aged in their early 70s [55]. The results showed a 35 g amount of wheat protein hydrolysate did not induce any increase in MPS despite containing 2.5 g of leucine. In contrast, a 60 g dose of wheat protein hydrolysate did significantly increase MPS, and this response was greater than a 35 g dose of whey protein (albeit not statistically significant), in which both of these beverages contained 4.4 g of leucine [55]. Circulating leucine concentrations were substantially lower when consuming only 35 g of wheat protein compared to a 60 g amount of wheat protein. Moreover, although plasma leucine concentrations were still lower after 60 g of wheat protein when compared to 35 g of whey protein (despite being matched for leucine content), it did appear to be substantially higher than when consuming a 35 g dose and was evidently enough to stimulate MPS to a similar degree to the whey protein group. While the 35 g dose of wheat contained 2.5 g of leucine (generally considered enough to maximally stimulate MPS), it is likely that this is insufficient to maximally stimulate MPS in older individuals, possibly due to decreased digestion and absorption of plant-based proteins in elderly individuals; therefore, they may require greater doses (e.g., 3–4 g) [78]. This demonstrates that older adults may require greater amounts of leucine to optimally stimulate MPS; therefore, a higher amount of overall plant-based protein, in this case wheat, may help facilitate more sustained availability of amino acids essential for maximizing MPS responses in older adults. More research on older adults with different types of plant-based proteins is required to support this contention.

More recently, investigations of MPS responses using non-animal protein sources have extended to fungi, in particular, mycoprotein, which is produced by continuous flow fermentation of the filamentous fungi *Fusarium venenatum*. Mycoprotein is a high-protein meat substitute derived from the fungus *Fusarium venenatum* (PTA 2684), and 100 g of its dry matter typically contains 45 g protein [79]. Initial work showed that a 70 g bolus of mycoprotein containing 31.5 g protein stimulated a greater increase in MPS than a leucine-matched (2.5 g) bolus of milk protein in young resistance-trained adults [63]. While the mycoprotein beverage in this study contained twice the amount of energy as the milk protein beverage, both beverages still provided adequate levels of protein previously shown to maximize MPS responses, thus canceling out protein quantity being a contributing factor to the results observed. The same group has also reported lower amounts of mycoprotein (~25–40 g), either as a protein concentrate [69] or as part of a blend with pea protein [68], to both significantly increase rates of MPS and to levels similar to the ingestion of isonitrogenous amounts of either 25 g protein from spirulina (blue-green alga) or chlorella (microalga) with exercise in healthy younger adults.

### 3.2. Effects of Whole-Food Protein Sources on Muscle Protein Synthesis Responses Between Vegan and Omnivorous Diets

It is critical to note that few studies have investigated MPS responses following the consumption of whole-food vegan sources. This certainly limits our understanding of whether the well-established higher fiber content and presence of anti-nutritional compounds in plant-based whole foods that can constitute a large part of vegan diets can compromise protein digestion and amino acid absorption kinetics and subsequently reduce MPS responses. Two studies from the Wall group incorporated mycoprotein as part of a whole-food vegan diet and compared the effects on MPS responses to an animal-based (whole-food) diet [65,66]. In the first of these two studies, 19 healthy female and male older adults were randomly assigned to either an omnivorous (consuming milk protein) or vegan (mycoprotein supplement) condition for three days, with both groups concomitantly performing resistance exercise [65]. The results showed both supplements significantly increased MPS in the exercised and resting legs, with no differences in the daily MPS responses observed between groups. In the second study, which builds upon the first one, 16 young adults in their early 20s were recruited. Participants adhered to a similar diet but with a higher protein content (1.8 g/kg) derived primarily from animal and mycoprotein sources. Concurrently with their daily unilateral leg resistance exercise, the participants proceeded to phase 2 of the study. During this phase, their protein intake increased to approximately 2 g/kg/d for 10 weeks, along with a progressive, high-volume (5 days/week) resistance-training program. The results of this study were consistent with the first one, showing a significant increase in daily myofibrillar protein synthesis rates, lean mass, thigh muscle volume, muscle fiber cross-sectional areas, and 1 RM strength, with no notable differences between groups [66].

To further determine the capacity for whole-food vegan protein sources to modulate MPS responses, Pinckaers and colleagues employed a cross-over design to investigate acute MPS responses to either 0.45 g of protein per kg body mass of either lean ground beef (with potatoes and string beans) or a plant-based meal (with a composition of quinoa, soybeans, chickpeas, and broad beans) in older (65–85 years) females and males [61]. Their results showed that ingestion of the whole-food meal containing beef induced a more rapid and greater rise in circulating plasma essential amino acids compared to the ingestion of an isonitrogenous and isocaloric whole-food meal containing only plant-based food sources (i.e., vegan) [61]. Furthermore, the greater postprandial amino acid availability following ingestion of the omnivorous meal resulted in ~47% higher muscle protein synthesis rates when compared to the ingestion of the vegan meal. The authors theorize that the disparate postprandial plasma amino acid profile following ingestion of both these meals can be attributed to differences in food digestibility [57], protein digestion and amino acid absorption [80], and splanchnic amino acid sequestration [81] following ingestion of the plant- versus animal-based protein sources. The significance of these findings is that they provide evidence to suggest that the food matrix specific to plant-based protein sources, including anti-nutritional factors (e.g., dietary fiber, trypsin inhibitors, and phytates), is significant enough to attenuate acute MPS responses. However, these differences in MPS responses were most recently shown to be absent when the dietary intervention was extended to a 10-day period [73]. Specifically, comparable rates of cumulative MPS responses were observed between isocaloric and isonitrogenous vegan and omnivorous diets providing 1.3 g/kg/day protein in community-dwelling older adults [73]. Such findings highlight the importance of incorporating free-living basal and postprandial MPS rates over an extended period of time with diet and physical activity. In this regard, while no specific exercise component was included in this study, participants were highly physically active, as evidenced by an average step count of ~12500 steps per day. Whether similar responses between vegan and omnivorous diets would be apparent if participants undertook resistance training (i.e., a more established anabolic stimulus that may increase dietary protein needs to facilitate higher MPS responses) remains an area for future investigation.

### 3.3. Limitations of Current Evidence Comparing Muscle Protein Synthesis Responses Between Vegan and Omnivorous Diets

Despite an increasing number of studies investigating the anabolic capacity of plant-based proteins and, by extension, whole-food diets on MPS responses, several methodological and technical restrictions still exist that limit current knowledge in this area. Firstly, comparatively fewer studies have measured cumulative MPS responses following strict vegan-based diets (and subsequent comparisons to omnivorous diets) over several days and weeks as opposed to acute MPS responses with single-source, supplemental proteins, predominantly powdered forms. In this regard, traditional acute measures of MPS in human skeletal muscle are largely confined to sterile (and costly) continuous intravenous infusions of amino acid tracers (i.e., phenylalanine) and serial blood sample collections. Such methods are further narrowed to short investigation periods (typically < 12 h) under tightly controlled laboratory conditions that not only prevent changes in protein turnover over days to weeks to be measured but also fail to capture the inherent effects of sleep patterns, medication, habitual or incidental forms of physical activity (and inactivity), and the cumulative effects of repeated exercise bouts that can all influence MPS responses [82,83]. Such limitations can be circumvented, to a large extent, by using/ingesting deuterium oxide labeling to quantify the cumulative rates of MPS over days and weeks. Indeed, the three studies to date that have compared the effects of vegan and omnivorous whole-food diets on MPS responses have utilized this methodological advance. However, outside of mycoprotein as a supplemental vegan protein source, there is a paucity of knowledge regarding the long-term effects (i.e., over weeks to months) of other supplemental vegan protein sources that can help form part of a whole-food vegan diet, such as, but not limited to, potato, pea, or corn protein, with multiple bouts of resistance exercise on MPS responses. Such knowledge would uncover whether vegan/plant protein sources (as part of a vegan diet) can be used as an effective means to build new muscle tissue at levels comparable to omnivorous diets in conjunction with resistance training across a human’s lifespan. It would also be interesting to determine how vegan and omnivorous diets alter the turnover rates of individual contractile, metabolic, and mitochondrial muscle proteins with exercise. Utilizing the proteomics-based Dynamic Proteome Profiling technique (with deuterium oxide labeling), we previously showed that short-term resistance exercise (three sessions over nine days) while consuming a high-fat, low-carbohydrate diet increased the synthesis of select myofibrillar and sarcoplasmic proteins compared to non-exercising participants consuming the same diet [84]. Whether vegan and omnivorous diets differentially alter specific muscle proteins with exercise to impact the magnitude of overall fractional and mixed MPS responses remains an area for future research.

## 4. The Gut–Muscle Axis: A Mechanism for Altering Muscle Anabolism Between Vegan and Omnivorous Diets?

Considering the inherent differences in quantities of macro- and micronutrients, dietary fiber, and presence of anti-nutritional factors between “typical” vegan and omnivorous diets, it stands to reason that some of the reported differences (or even similarities) in MPS responses between these dietary patterns may be mediated via the microbiota–gut–muscle axis. Diet-induced changes in the gut microbiome through carbohydrate and protein fermentation and intestinal inflammation [39,85] typically manifest within 24 h [86]. As such, given the changes are in alignment with the time course of diet-induced changes in MPS responses, alterations in microbiome responses may impact the magnitude of MPS responses with diet and/or exercise. As no study to date has investigated and compared the effects of omnivorous and vegan diets on concomitant MPS and microbiome responses, the following discussion provides a theoretical mechanistic basis for how each dietary pattern may influence the gut–muscle axis.

Perhaps the most logical starting point is the different macronutrients and other dietary components that vary between typical vegan and omnivorous dietary patterns. Western-style omnivorous diets tend to be higher in saturated and overall fat, protein, and red meat, all of which have been associated with compromised gut barrier function and/or elevated inflammatory markers [87,88]. High intakes of saturated and overall fat have been shown to disrupt tight junction proteins in the intestinal epithelium in mice [89], while increased bile secretion in response to high-fat diets may influence intestinal barrier function through receptor-dependent and receptor-independent mechanisms, increase intestinal inflammation, and cause shifts in gut microbiota composition detrimental to the gut barrier [87]. A higher intake of heme iron, common in red meat, may similarly lead to shifts in the gut microbiota that promote degradation of the mucin-2 network and, subsequently, the mucus bi-layer in the colon, also increasing gut permeability [90]. Each of these serves to promote the translocation of microbial products, such as lipopolysaccharides from Gram-negative bacteria into the systemic circulation, which leads to low-grade inflammation [91]. Moreover, a higher protein intake has been shown to favor a pro-inflammatory microbiota profile, as well as compromise the colonic epithelium structure, likely due to increased fermentation products resulting from the microbial metabolization of aromatic and sulfur-containing amino acids (though this may be offset if there is increased consumption of dietary fiber) [92]. The integrity of the gut barrier and the inflammatory nature of these dietary components is important to consider, as systemic inflammation as a result of this may interfere with insulin sensitivity [93] and the mechanistic target of rapamycin complex 1 (mTORC1) activation [18]. Nonetheless, the higher overall protein and essential amino acid content typical of omnivorous diets may mitigate many of these adverse effects, ultimately supporting MPS despite the potential suboptimal microbiota responses discussed.

**Figure 1:**
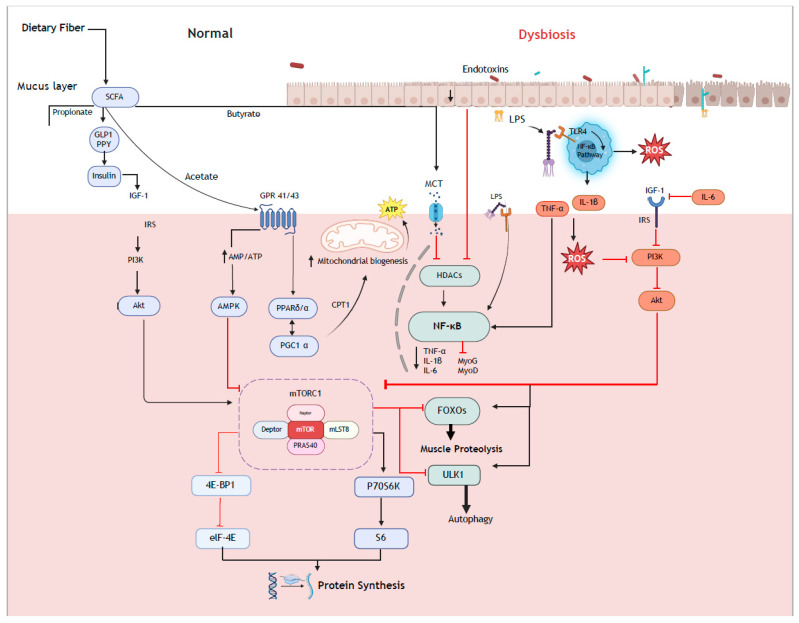
Putative cellular mechanism that may regulate the muscle protein synthesis responses via the gut–muscle axis. The higher dietary fiber intake typically associated with vegan diets may mediate increases in the production of short-chain fatty acids that, through activation of the IGF-mTORC1 signaling axis, can stimulate muscle protein synthesis responses.

Conversely, omnivorous diets are typically lower in fiber when compared with vegan diets, which may reduce intestinal SCFA production. Mechanistically, this can increase both Forkhead box O (FOXO) and nuclear factor–kappa B (NF-κB) signaling pathways (Figure 1) and, subsequently, muscle protein breakdown via the regulation of E3 ubiquitin ligases and specific autophagy factors [10]. However, while the higher fiber content of vegan diets may enhance intestinal SCFA production and subsequently reduce FOXO and NF-κB signaling, higher levels of SCFAs may also activate AMP-activated protein kinase (AMPK) signaling. AMPK is a cellular energy sensor that is activated when cellular energy levels are low and subsequently restores energy balance by both promoting catabolic processes that generate ATP and inhibiting anabolic processes that consume ATP. One mechanism by which AMPK mediates this role is through inhibiting mTORC1 signaling through direct and indirect phosphorylation events [94]. The phosphorylation and activation of mTORC1 through exercise and nutrition is a key determinant for increasing rates of MPS by regulating translation initiation via the phosphorylation of downstream ribosomal protein S6 kinase (S6K) and eukaryotic translation initiation factor 4E-binding proteins (4E-BPs) [95]. Although moderate AMPK activation generally benefits overall metabolic health and does not necessarily negate robust anabolic stimuli, sustained or excessive AMPK activity could blunt the phosphorylation of mTORC1’s downstream targets and thus hinder maximal rates of MPS. Further to this, vegan dietary patterns can also present challenges with respect to lower intakes of certain essential amino acids that are crucial for the robust stimulation of mTORC1. This may be compounded by reductions in protein digestibility, absorption, and bioavailability due to the presence of insoluble fiber and anti-nutrient factors, such as phytates and oxalates.

The production of SCFAs from dietary fiber fermentation is proposed as a key mechanism of action to influence muscle mass and function via the microbiota–gut–muscle axis. The metabolite butyrate appears to be one of the main intermediaries of gut and muscle through its direct (i.e., regulation of anabolic pathways) and indirect actions (i.e., anti-inflammatory processes, immune regulation, and homeostasis maintenance). Key bacteria that degrade carbohydrates into SCFAs belong mostly to Firmicutes phylum, including *Bacteroides* spp., *Prevotella* spp., *Ruminococcus* spp., *Bifidobacterium* spp., *Clostridium* spp., and *Akkermansia muciniphila*. Compared to omnivorous diets, both short- and long-term vegan diets resulted in higher levels of *Faecalibacterium prausnitzii*, *Roseburia* spp., *Clostridium* spp., and *Anaerostipes* spp., which are well-known SCFA producers, especially butyrate. However, given only a small proportion of SCFAs are present within a fecal sample, it is often difficult to confirm this relationship via fecal analysis. Notwithstanding, the creation of next-generation probiotics that are known butyrate and other SCFA producers might be promising not only as an ergogenic aid in exercise-induced muscle adaptation but may also mitigate the onset and progression of diseases characterized by muscle loss, such as sarcopenia and cancer cachexia.

A less-explored mechanism by which microbes might interact with muscle health is through their role in direct protein metabolism, particularly in plant-based diets. Long-term adherence to plant-based diets could conceivably promote microbial adaptations that influence colonic protein metabolism, although the extent to which this meaningfully contributes to amino acid availability remains speculative. While protein digestion primarily occurs in the small intestine, plant proteins are generally less digestible than animal proteins, which may lead to a greater delivery of undigested protein to the colon when consumed in large amounts. Over time, microbial shifts in response to plant-based diets may facilitate colonic amino acid and peptide absorption, potentially compensating for lower small intestinal digestibility. However, as discussed earlier, colonic protein fermentation also produces potentially harmful byproducts, such as p-cresol, ammonia, and hydrogen sulfide, which may have negative implications for gut and systemic health. Thus, while microbial adaptations could enhance colonic amino acid salvage, an increased delivery of plant-based protein to the colon may not always be favorable.

Beyond the role of the gut microbiota, dietary microbes present in fermented foods or probiotics may also influence protein metabolism and muscle health. Although not traditionally considered part of the gut–muscle axis, microbial fermentation of plant-based proteins prior to ingestion or during early digestion may improve their availability by reducing anti-nutritional factors and enhancing protein hydrolysis, thereby increasing upper-intestinal amino acid absorption. Studies have shown that probiotic supplementation alongside plant-based proteins can enhance amino acid bioavailability in the small intestine and lead to improvements in body composition over time [96,97]. Given that these effects were observed after two weeks of supplementation, it remains unclear whether this represents an adaptive response to microbial colonization or an acute enhancement of early protein metabolism, though in vitro models suggest the latter is more likely [98]. Future research could explore how the microbial fermentation of foods before ingestion or inclusion of probiotics in supplements—by increasing protein solubility and hydrolysis—may serve as a practical strategy to optimize muscle health in plant-based diets.

In practice, both vegan and omnivorous diets can support muscle health when care is taken to optimize dietary quality. Excessive consumption of red and processed meats may raise the inflammatory burden and disrupt gut function, negating some of the potential benefits of complete proteins and a higher leucine content. Well-planned omnivorous diets, such as Mediterranean-style eating patterns that emphasize lean proteins, fiber-rich plant-based foods, and healthy fats, can mitigate these risks by reducing inflammation and promoting a favorable gut microbiome. Meanwhile, carefully designed vegan diets with diverse protein sources, adequate total protein intake, and methods to enhance nutrient bioavailability can leverage the anti-inflammatory potential of fiber and phytonutrients. Striking the right balance between nutrient supply, gut health, and metabolic signaling ultimately appears central to maximizing muscle protein synthesis and preserving skeletal muscle function.

## 5. Conclusions and Future Directions

The mechanisms linking the gut–muscle axis to dietary patterns remain poorly understood in humans, with much of the current evidence extrapolated from animal models or theoretical pathways. While SCFAs, systemic inflammation, and signaling pathways, like mTORC1 and AMPK, are promising areas of focus, there is a need for human studies that directly connect microbiota-derived metabolites to muscle protein synthesis and breakdown. Advanced techniques, such as isotope tracer studies and metabolomics, could provide insight into these mechanisms. Additionally, the role of exercise as a modulator of gut microbiota and its interaction with diet is underexplored. The current evidence indicates that vegan diets can effectively stimulate rates of MPS to levels comparable to omnivorous-based supplements and whole-food diets. However, further interrogation of chronic MPS responses (i.e., over multiple weeks to months) with increasingly popular plant-based protein supplements (that form part of a balanced, whole-food vegan diet), such as potato, corn, and oats, are still required. Whole-food diets, with their complex food matrices and bioactive compounds, may exert unique effects on gut microbiota, nutrient bioavailability, and MPS that cannot be captured by studying isolated proteins. Future research should explore the effects of complete dietary patterns, particularly in older adults and athletes with higher protein demands. Additionally, whether specially formulated hydrolyzed or fermented versions of plant-based sources to improve protein digestibility and absorption can induce higher rates of MPS represents a further area for research. Future research should also prioritize multi-center, long-term studies across diverse populations to account for geographic variations in dietary cuisines and cooking practices, particularly when it comes to the preparation of different plant-based meals that form meal components of vegan diets, to help improve the broader relevance of its findings. Studies examining how resistance (and endurance) exercise influence gut microbiota composition and function and whether these changes synergize with dietary interventions to enhance muscle adaptations are crucial. Finally, lifestyle factors, such as stress, sleep, and medication use, which can significantly impact gut microbiota composition, are rarely considered in the current research. Addressing these variables could provide a more holistic understanding of how diet and lifestyle collectively influence the gut–muscle axis. By integrating these approaches with long-term dietary interventions, future research could develop tailored strategies to improve muscle outcomes across diverse populations and life stages and help bridge critical gaps in our understanding of the gut–muscle axis, guiding evidence-based dietary recommendations for health and performance.

## Figures and Tables

**Table 1:** Exploratory human studies describing correlations/interactions between short- and long-term dietary habits, gut microbiota, and associated metabolites.

Author, Country (PMID)	Participant Demographic (N, Male% and Age M ± SD)	Dietary Groups	Minimum Duration of Diet	Dietary Analysis	Method of Gut Microbiota and Metabolomic Assessment	Composition of Gut Microbiota—Main Findings
Reddy et al., 1975 ([24]) USA (1138032)	N = 8 Sex: Both but not specified Age: 30–50 yrs	Habitual mixed Western diet (n = 8) Non-meat diet (n = 8)	No details for Western diet. Four-week intervention after consuming habitual Western diet.	NA	One stool sample per person for 4 consecutive days. Only one sample used for microbial analysis. Traditional plate count culture.	Total anaerobic microflora as well as counts of *Bacteroides*, *Bifidobacterium*, *Peptococcus*, and anaerobic *Lactobacillus* were significantly higher during high meat, mixed Western diet compared to non-meat diet period; Increased *Streptococcus* and *Staphylococcus* and decreased *Bacillus* following non-meat diet; Reduced diphtheroides and enterobacteria following non-meat diet and mixed Western diet, respectively.
Zimmer et al., 2012 ([25]) Germany (21811294)	N = 498 Sex: Vegan 43%, Vegetarian 34%, Omnivore 38% Age: Vegan 49.2 ± 14.6 yrs, Vegetarian 56.7 ± 15.1 yrs, Omnivore 53.7 ± 14.8 yrs	Vegan (n = 105) Vegetarian (n = 144) Omnivore (n = 249)	Not specified for each group (≥one month inclusion criteria).	Short questionnaire that included specific food intake.	One stool sample per person. Viable bacterial cell counts via agar plates.	Vegetarians had significant lower microbial counts of *Bacteroides* and *Bifidobacterium* species compared to omnivores (control group 1); No difference in *E. coli Biovare*, *Klebsiella*, *Enterobacter*, *Enterococcus*, *Enterobacteriaceae*, *Lactobacillus*, *Citrobacter*, and *Clostridia* species between vegetarians and omnivores; Vegan diet had significantly lower *Bacteroides*, *Bifidobacterium*, *E. coli*, and *Enterobacteriaceae* species compared to omnivores (control group 1); No significant differences in *Enterobacter*, *Enterococcus*, *Clostridium*, *Klebsiella*, *Lactobacillus*, and the total viable count between vegans and omnivores.
Kabeerdoss et al., 2012 ([26]) India (22182464)	N = 56 Sex: 0% Age *: Vegetarian: 19 yrs (18–19) Omnivore: 19 yrs (18–20)	Lacto-vegetarian (n = 32) Omnivore (n = 24)	Not specified.	24 h diet recall and FFQ (3 months).	One stool sample per person. 16srRNA sequencing (variable region not specified).	Omnivorous group displayed higher levels of *C. coccoides*–*E. rectale* group (*Clostridium* cluster XIVa bacteria), specifically *Roseburia*–*E. rectale* compared to vegetarians; No increase in *R. productus*–*C. coccoides* and *Butyrivibrio*.
Matijasic et al., 2014 ([27]) Slovenia (24173964)	N = 60 Sex: Vegan 60%, Lacto-vegetarian 58.3%, Vegetarian 59.4%, Omnivore 43.3% Age **: Vegan 35 (2–63) Lacto-vegetarian 34 (30–67) Vegetarian 35 (2–67) Omnivore 30 (1.5–61)	Vegan (n = 20) Lacto-vegetarian (n = 11) Vegetarian (n = 31) Omnivore (n = 29)	Not specified for each group (≥12 months inclusion criteria).	In-house questionnaire including consumption of particular foods.	One stool sample per person. PCR-DGGE finger printing of 16S rRNA V3 region.	Vegetarian diet displayed higher ratio of *Bacteroides*–*Prevotella* but lower *C*. *coccoides* group (*Clostridium* cluster XIVa) compared to omnivores; Vegetarians also displayed higher ratio of *B. thetaiotaomicron* and *C. clostridioforme*; *C. clostridioforme* and *F. prausnitzii* ratios were higher in vegans; DGGE analysis showed higher *Bifidobacterium*, *Streptococcus*, *Collinsella*, and *Lachnospiraceae* in omnivores compared to vegetarians; however, *Subdoligranulum* was higher among vegetarians.
Ferrocino et al., 2015 ([28]) Italy (26035837)	N = 153 Sex: 35% in Ovo-lacto-vegetarian, 45% in both Vegan and Omnivore Age: Ovo-lacto-vegetarian (39.0 ± 9.0) Vegan (37.0 ± 10.0) Omnivore (37.0 ± 9.0)	Ovo-lacto-vegetarian (n = 51) Vegan (n = 51) Omnivore (n = 51)	Not specified for each group (≥12 months inclusion criteria).	Not stated.	One stool sample per person. Ribosomal RNA Denaturing Gradient Gel Electrophoresis (rRNA-DGGE).	No significant difference in microbiota diversity between diet groups, though geographical origins were different. In V3 region analysis, omnivores showed presence of bands identified as *B. salanitronis*, *B. coprocola*, and *P. copri*, while vegetarians displayed *P. micans* and *B. vulgatus*; Vegans displayed *B. salyersiae*. In the V9 region, omnivores showed presence of bands identified as *E. coli*, vegetarians displayed *F. prausnitzii*, and vegans displayed *V. parvula*; Plate count analysis revealed lower *Coliform* and *Bifidobacterium* counts in vegans, while omnivores showed reduced *Bacteroides* and *Prevotella* but higher *B. fragilis* compared to other two groups.
De Filippis et al., 2016 ([29]) Italy (27769291)	N = 153 Sex: 35% Ovo-lacto-vegetarian, 45% in both Vegan and Omnivore Age: Ovo-lacto-vegetarian (39.0 ± 9.0) Vegan (37.0 ± 10.0) Omnivore (37.0 ± 9.0)	Ovo-lacto-vegetarian (n = 51) Vegan (n = 51) Omnivore (n = 51)	Not specified for each group (≥ 12 months inclusion criteria).	7-day weighed food diary.	One stool sample per person. 16S rRNA gene sequencing (V1-V3 region).	No significant difference in microbiota diversity between diet groups. Correlations between *Roseburia*, *Lachnospira*, and *Prevotella* and vegetable-based diets (negative with omnivorous diets); *L-Ruminococcus* and *Streptococcus* were positively associated with diets of animal origin and negatively with plant-based diets. Please note that this study factored in adherence to Mediterranean diet across all three diet groups, and this could influence results.
Wu et al., 2016 ([30]) USA (25431456)	N = 31 Sex: Not specified Age: Not specified (18–40 yrs)	Vegan (n = 15) Omnivore (n = 16)	Not specified for each group (≥ six months inclusion criteria).	3 × 24 h dietary recalls.	One stool sample per person. 16S rRNA sequencing (V1 and V2 regions). Metabolite profiling (LC-MS and GC-MS).	No significant differences in diversity, composition, or bacterial abundance were found between groups *. Please note that sample size is very small; thus, caution should be used when interpreting these findings.
Losasso et al., 2018 ([31]) Italy (29556222)	N = 101 Sex: Vegetarian (30%), Vegan (34.7%), Omnivore (26.7%) Age: Vegetarian: 42.3 ± 13.2 yrs Vegan: 39.4 ± 11.1 yrs Omnivore: 45.0 ± 13.9 yrs	Vegetarian (n = 32) Vegan (n = 26) Omnivorous (n = 43)	Not specified for each group (≥ 12 months inclusion criteria).	14-day semi-quantitative FFQ. 24 h recall.	One stool sample per person. 16S rRNA sequencing (V3 and V4 regions).	Microbiota diversity higher in vegetarians compared to omnivores; No significant difference in composition (beta diversity); Both vegetarian and vegan groups demonstrate higher counts of Bacteroidetes compared to omnivores; No significant difference in Firmicutes/Bacteroides ratio distribution among the three groups or in *Prevotellaceae* abundance distribution.
Trefflich et al., 2021 ([32]) Germany (34073495)	N = 72 Sex: 50% for both Vegan and Omnivore Age: Vegan: 37.5 yrs Omnivore: 38.5 yrs	Vegan (n = 36) Omnivorous (n = 36)	Vegan: 4.8 yrs. Omnivore: No detail.	3-day weighed food diary.	One stool sample per person. 16S rRNA sequencing (V3 and V4 regions). Metabolite profiling (GC-MS).	Microbiota diversity (Shannon only) higher in omnivores than in vegans on species level; No difference in composition (beta diversity) between groups; Vegans had higher abundance of Tenericutes phylum and several bacterial species, including *B. desmolans* and *C. colinum*, while omnivores showed higher levels of *B. uniformis* and *B. vulgates*, *P. excrementihominis,* and *D. invisus.*
Prochazkova et al., 2022 ([33]) Czech Republic (35071294)	N = 95 Sex: Vegan (60%), Omnivore (48%) Age: Vegan: 30.9 ± 10.5 yrs Omnivore: 31.3 ± 11.2 yrs	Vegan (n = 62) Omnivore (n = 33)	Not specified for each group (≥ 36 months inclusion criteria).	3-day prospective dietary record supervised by trained dietitian.	One stool sample per person. 16S rRNA sequencing (V4 region). Metabolite profiling (NMR and LC/GC-MS).	Higher microbiota diversity in omnivores compared to vegans. Significant differences in bacteria composition (beta diversity) between diet groups; Enrichment of *Lachnospira*, *Lachnospiraceae* NK4A136 group, and *Ruminiclostridium* and depletion of *Alistipes*, *Bifidobacterium*, *Blautia*, *Fusicatenibacter*, *Dorea*, *Anaerostipes*, and Ruminococcaceae uncultured in vegans compared with omnivores.
Stege et al., 2022 ([34]) Netherlands (35115599)	N = 149 Sex: Vegetarian (32%), Vegan (34%), Pescatarian (36%), Omnivore (32%) Age ***: Vegetarian 45 yrs Vegan 37 yrs Pescatarian 51 yrs Omnivore 47 yrs	Vegetarian (n = 34) Vegan (n = 32) Pescatarian (n = 33) Omnivore (n = 50)	Not specified for each group (≥ six months inclusion criteria).	None.	One stool sample per person. Metagenomic shotgun sequencing. ResCap sequencing (resistome analysis).	No significant difference in microbiota diversity between all diet groups. Composition (beta diversity) was also similar between groups; Vegans displayed lower abundance of *R. torques*, *S. thermophilus*, *Clostridium* sp., *C. phoceensis,* and *C. saccharolyticum* compared to omnivores; Compared to pescatarians, vegans displayed lower abundance of *S. thermophilus*, *L. lactis*, and *Firmicutes bacterium* CAG:313; Omnivores displayed higher abundance of *L. delbrueckii*, *C. comes*, *D. formicigenerans*, *D. longicatena*, *L. asaccharolyticus*, and *Phascolarctobacterium* CAG:266 compared to vegans.
Tarallo et al., 2022 ([35]) Italy (34315772)	N = 120 Sex: 40% in each group Age: Vegetarian (40.6 ± 11.7) Vegan (39.1 ± 11.6) Omnivore (40.5 ± 13.2)	Vegetarian (n = 40) Vegan (n = 40) Omnivore (n = 40)	Vegetarian: 9.7 ± 9.0 yrs Vegan: 8 ± 2.5 yrs	FFQ.	One stool sample per person. Metagenomic shotgun sequencing.	Higher relative abundance of *P. copri* and *Roseburia* sp. CAG 182 in vegans/vegetarians compared to omnivores. Higher relative abundance of *B. dorei* (currently *P. dorei*) in omnivores compared to vegans/vegetarians.
Seel et al., 2023 ([36]) Germany (37111133)	N = 258 Sex: 38% Omnivore 20% Flexitarian 28% Ovo-lacto-vegetarian 29% Vegan Age *: Omnivore 33.0 (17.0) Flexitarian 29.5 (16.8) Ovo-lacto-vegetarian 28.0 (14.0) Vegan 25.0 (9.8)	Ovo-lacto-vegetarian (n = 65, n = 51 final), Vegan (n = 58, final 52), Flexitarian (n = 70, n = 52 final), Omnivore (n = 65 screening, n = 51 final)	Ovo-lacto-vegetarian 6.0 ± 10.0 yrs, Vegan: 3.0 ± 3.0 yrs, Flexitarian: 8.0 ± 17.8 yrs, Omnivore 32.0 ± 20.0 yrs.	5-day self-reported dietary record.	One stool sample for each participant. 16S rRNA gene sequencing (V3-V4 region).	No significant difference in microbiota diversity between all diet groups. Significantly different between omnivore and vegan diets in bacterial richness (when directly compared statistically); Vegans and vegetarians significantly differed in microbiota composition (beta diversity) compared to omnivores; Low abundance of *Bacteroides*, *Lachnospiraceae*_1, *Butyricoccus*, *Lachnospiraceae* UCG_004, and *Haemophilus* in omnivores (highest in vegans); Low abundance of *Dorea*, *Ruminococcus torques* group, *E. ruminantium* group, Ruminococcaceae, *Lachnospiraceae*_2, *Lactobacillus*, and *Senegalimassilia* (highest in omnivores); Low abundance of *Eubacterium siraem* group in vegetarians (highest in vegans).
Fackelmann et al., 2025 ([37]) Italy, UK, and USA (39762435)	N = 21,561 Sex: Not included Age: 52 ± 12.5 yrs	Vegetarian (n = 1088), Vegan (n = 656), Omnivorous (n = 19,817)	Not specified for each group (≥ 12 months inclusion criteria).	Food Frequency Questionnaire.	One stool sample per person. Metagenomic shotgun sequencing.	Lower microbiota diversity in vegans and vegetarians compared to omnivores. Significant differences in composition (beta diversity) between diet groups; Omnivores were characterized by meat-associated bacteria (*R. torques*, *B. wadsworthia*, and *A. putredinis*), while vegans showed higher abundance of beneficial bacteria (*Lachnospiraceae*, *Butyricicoccus* sp., and *R. hominis*); Higher *S. thermophilus* in omnivores and vegetarians compared to vegans.

Abbreviations: N, Number of Participants; M, Mean; SD, Standard Deviation; yrs, Years; FFQ, Food Frequency Questionnaire; PCR-DGGE, Polymerase Chain Reaction-Denaturing Gradient Gel Electrophoresis; rRNA, Ribosomal Ribonucleic Acid; LC-MS, Liquid Chromatography–Mass Spectrometry; GC-MS, Gas Chromatography–Mass Spectrometry; NMR, Nuclear Magnetic Resonance. * Median (interquartile range); ** Median (range); *** Median values.

**Table 2:** Human studies describing dietary interventions on the gut microbiota composition and associated metabolites.

Author, Country (PMID)	Participant Demographic (N, Male% and Age M ± SD)	Dietary Groups	Study Design and Intervention Duration	Dietary Analysis	Method of Gut Microbiota and Metabolomic Assessment	Composition of Gut Microbiota—Main Findings
van Faassen et al., 1987 ([38]) Netherlands (3120571)	N = 12 Sex: 100% Age: Not specified, age range 20–27 yrs	Mixed Western (n = 12), Lacto-ovo-vegetarian (n = 12), Vegan (n = 12) in cross-over design Mixed Western: 38% fat, 17% protein. Lacto-ovo-vegetarian: 32% fat, 16% protein (0% meat. Vegan: 33% fat, 17% protein. All meals were provided in a controlled metabolic ward setting in randomized order.	Cross-over study design. 20 days for each diet period, with an 8-day adaptation period on mixed diet first. No details regarding washout length provided.	NA	Two stool samples collected per person. Traditional plate count (Agar- based culturing).	Both vegan and lacto-ovo-vegetarian diets reduced *clostridia* counts; Vegan diet also resulted in reduced *lactobacilli* and *enterococci* compared to lacto-ovo-vegetarian diet; Mixed Western diet increased *lactobacilli* compared to vegan diet.
David et al., 2014 ([39]) USA (24336217)	N = 11 (n = 10 per diet; 9 individuals completed both diet interventions) Sex:55% Age: 28.1 ± 3.72 yrs	Plant-based diet (n = 9). Animal-based diet (n = 9). Animal-based diet: dietary fat 69.5 ± 0.4% kcal, dietary protein 30.1 ± 0.5% kcal, and dietary fiber ~0%. Plant-based diet: dietary fiber 25.6 ± 1.1 g/1000 kcal, dietary fat 22.1 ± 1.7% kcal, and dietary protein 10.0 ± 0.3% kcal.	Cross-over study design. Five-day controlled diet intervention with meals provided. Six-day wash out period.	NA	Daily stool samples from each participant. 16S rRNA gene sequencing (V4 region).	Microbiota diversity did not change significantly on either diet, but composition (beta diversity) significantly increased following the animal-based diet; Animal-based diet significantly increased bile-tolerant microorganisms (*Alistipes*, *Bilophila*, and *Bacteroides*) while decreasing Firmicutes that metabolize plant polysaccharides (*Roseburia*, *E. rectale*, and *R. bromii*); Both diets showed rapid (within 24 h) and reproducible effects, with RNA-seq revealing expression differences between carbohydrate and protein fermentation pathways. Animal-based diet increased *B. wadsworthia* abundance and activity.
Zhang et al., 2018 ([40]) China, Sweden, and USA (29755475)	N = 29 Sex: 47% Omnivore to lacto-ovo-vegetarian 71% Long-term omnivore 43% Long-term vegetarian Age: Omnivore to lacto-ovo-vegetarian 35.4 ± 9.8 yrs Long-term omnivore 35.7 ± 10.3 yrs Long-term vegetarian 33.7 ± 5.8 yrs	Study groups: Omnivore to lacto-ovo-vegetarian (n = 15); Control 1: long-term omnivore (n = 7); Control 2: long-term vegetarian (n = 7). Study group changed from omnivorous to lacto-ovo-vegetarian diet for 3 months (self-selected foods, no meals provided). Control Group 1: Maintained regular omnivorous diet (no dietary changes required). Control Group 2: Long-term vegetarians maintained their lacto-ovo-vegetarian diet (no dietary changes required).	Single arm, unblinded study design. 3-month diet change from omnivore to lacto-ovo-vegetarian. The other 2 groups were monitored during that time and continued their usual diet (long-term omnivore and vegetarian).	3-day weighted food diary and dietary questionnaire.	One stool sample per person at days 0 and 91. Metagenomic shotgun sequencing.	No significant difference in gene count or diversity at all levels between the two time points in any groups; Using Jensen–Shannon divergence, a trend for reduction after changing to the short-term vegetarian diet, reaching significance at the genus and species level; *Alistipes* significantly reduced when switching to lacto-ovo-vegetarian, along with unclassified *Alistipes* sp. HGB5, *A. shahii*, and *A. putredinis. B. finegoldii* enriched in the omnivores, while *P. duerdenii* and *C. symbiosum*, *B. hydrogenotrophica* (formally known as *R. hydrogenotrophicus*) were increased in long-term vegetarians.
Kohnert et al., 2021 ([41]) Germany (33807447)	N = 53 Sex: 31% Vegan, 44% Meat-rich Age: Vegan: 33.2 ± 11.2 Meat-rich: 29.9 ± 9.5	Meat-rich diet (>150 g of meat per day, n = 27). Strict vegan (n = 26). Every participant received extensive training on his/her assigned diet and detailed written information, including a recipe book. No meals were provided, and participants were free to choose their food within their assigned diet.	Monocentric, controlled, randomized trial with a parallel group design. Four-week intervention.	NA	Two stool samples were collected at the beginning and at the end of the trial. 16S rRNA gene sequencing (V4 region).	No significant change in species diversity and composition (alpha and beta diversity) following diet intervention; Increase and decrease in *Coprococcus* with vegan and meat-rich diets, respectively. Amplicon sequence variants of genera revealed enrichment of *Alistipes*, *Bacteroides*, *Blautia*, *Coprococcus*, *Dialister*, *Dorea*, *Faecalibacterium*, *Phascolarctobacterium*, and *Ruminococcus* and depletion of *Akkermansia*, *Bacteroides*, *Bifidobacterium*, *Clostridium*, *Coprococcus*, *Faecalibacterium*, *Roseburia*, and *Ruminococcus* following vegan diet; Following meat-rich diet, amplicon sequence variants of genera revealed enrichment of *Alistipes*, *Bacteroides*, *Blautia*, *Clostridium*, *Faecalibacterium*, *Megamonas*, *Roseburia*, and *Ruminococcus* and depletion of *Bacteroides*, *Bifidobacterium*, *Blautia*, *Dialister*, *Faecalibacterium*, *Gemminger*, *Phascolarctobacterium*, *Prevotella*, *Ruminococcus*, and *Sutterella*; Bacteria with significant interaction in terms of diet and time changed significantly in meat-rich diet over time compared to their respective changes in vegan diet, including enrichment of *Bacteroides*, *Clostridium*, *Faecalibacterium*, and *Roseburia* following meat-rich diet, while they were depleted following vegan diet. Conversely, *Bacteroides*, *Blautia*, *Dialister*, *Faecalibacterium*, and *Ruminococcus* were depleted following meat-rich diet but enriched following vegan diet.

Abbreviations: N, Number of Participants; M, Mean; SD, Standard Deviation; yrs, Years; rRNA, Ribosomal Ribonucleic Acid.

**Table 3:** Muscle protein synthesis (MPS) responses to plant-based and omnivorous supplement and whole-food dietary protein sources.

Study (PMID)	Cohort Details	Vegan Protein Source	Comparison Protein Source	Exercise	Key Findings
Wilkinson et al., 2007 ([53]) (17413102)	8 healthy men (21.6 ± 0.3 y; mean ± SEM)	Isonitrogenous, isoenergetic, and macronutrient-matched soy beverage (18 g protein, 750 kJ)	Isonitrogenous, isoenergetic, and macronutrient-matched milk beverage (18 g protein, 750 kJ)	Unilateral leg press, hamstring curl, and knee extension: 3 × 10 reps @ 80% of 1 RM + 1 × AMRAP of each exercise	MPS greater after milk (0.10 ± 0.01%/h) than soy (0.07 ± 0.01%/h; *p* = 0.05).
Tang et al., 2009 ([15]) (19589961)	3 × 6 groups of resistance-trained young males (22.8 ± 3.9 y; mean ± SD)	Soy (22.2 g) protein dissolved in 250 mL water with sucralose (1 g Splenda)	Whey (21.4 g) OR Casein (21.9 g) protein dissolved in 250 mL water with sucralose (1 g, Splenda)	Unilateral leg press and knee extension (4 × AMRAP @ 10- to 12-RM)	Mixed MPS after consumption of whey was ∼93% greater than casein (*p* < 0.01) and ∼18% greater than soy (*p* = 0.067); Similar result observed after exercise (whey > soy > casein); MPS following whey consumption was ∼122% greater than casein (*p* < 0.01) and 31% greater than soy (*p* < 0.05); MPS was greater with soy consumption at rest (64%) and following resistance exercise (69%) compared with casein (*p* < 0.05).
Yang et al., 2012 ([54]) (22698458)	30 older males (age 71 ± 5 y; mean ± SD)	20 g or 40 g soy protein isolate (S20 and S40)	0 g, 20 g, or 40 g of whey protein isolate (W20 and W40)	Unilateral knee-extensor exercise (3 × AMRAP @ 10 RM)	MPS for S20 was less than W20 (*p* = 0.02); MPS not different from 0 g (*p* = 0.41) in both exercised and non-exercised leg muscles; For S40, MPS reduced compared with W40 under both rested and post-exercise conditions (both *p* < 0.005); S40 increased MPSgreater than 0 g under post-exercise conditions (*p* = 0.04).
Gorissen et al., 2016 ([55]) (27440260)	60 healthy older men (71 ± 1 y; mean ± SEM)	35 g wheat protein (n = 12), 35 g wheat protein hydrolysate (WPH-35; n = 12), or 60 g wheat protein hydrolysate (WPH-60; n = 12)	35 g micellar casein (MCas-35; n = 12), 35 g whey protein (Whey-35; n = 12), hydrolysate (WPH-60; n = 12)	No exercise	Myofibrillar protein synthesis rates increased after ingesting micellar casein-35 (*p* < 0.05); Myofibrillar protein synthesis rates were higher after ingesting MCas-35 than after wheat protein hydrolysate-35 (*p* < 0.05); Ingestion of WPH-60 increased myofibrillar protein synthesis rates above basal rates (*p* < 0.05).
Oikawa et al., 2020 ([56]) (32349353)	24 healthy females (20 ± 1 y; mean ± SD)	Potato protein (25 g providing 1.6 g/kg body mass per d total protein)	Control (0.8 g/kg body mass per d total protein)	3 d/wk unilateral leg press and extension: 6 sets @30% 1-RM	MPS increased in potato protein group above baseline both at rest and post-exercise (*p* < 0.05); Control group showed MPS increase only post-exercise (*p* < 0.05); Similar exercise-induced MPS between groups.
Pinckaers et al., 2021 ([13]) (33597056)	36 healthy young males (23 ± 3 y; mean ± SD)	30 g wheat protein (WHEAT)	30 g milk protein (MILK) OR 30 g blend combining 15 g wheat plus 15 g milk protein (WHEAT + MILK)	No exercise	Ingestion of protein increased myofibrillar protein synthesis rates in all treatments (*p* < 0.05); Postprandial myofibrillar protein synthesis rates did not differ between MILK v. WHEAT or MILK v. WHEAT + MILK.
Pinckaers et al., 2022 ([57]) (35438672)	24 healthy young males (24 ± 4 y; mean ± SD)	30 g potato-derived protein	30 g milk protein	Unilateral leg press (3 × 8 reps @ ~80% 1-RM + AMRAP) + unilateral seated knee extension machine (3 × of 8 reps @ ~80% 1-RM + AMRAP)	Ingestion of both potato and milk protein increased mixed muscle protein synthesis rates when compared with basal postabsorptive values or post-exercise values (*p* < 0.05); No differences between treatments.
Kouw et al., 2022 ([58]) (34881688)	24 men (age 24 ± 5 y; mean ± SD)	40 g of protein as a lysine-enriched wheat and chickpea protein product (plant, n = 12)	40 g chicken breast fillet (chicken, n = 12)	No exercise	Ingestion of both plant and chicken increased muscle protein synthesis rates from post-absorptive to postprandial (*p* < 0.05); No differences between plant and chicken.
Davies et al., 2022 ([59]) (36145064)	16 healthy adults (age = 25 ± 5; mean ± SD)	0.33 g·kg fava bean protein (FBP)	0.33 g·kg control (CON) beverage (EAA-free mixture)	6 × 10 maximal effort unilateral (dominant limb) isokinetic knee extensor contractions (concentric and eccentric) at a velocity of 90°·s^−1^	Resting Limb: No difference between FBP and CON (myofibrillar protein synthesis did not increase in response to ingestion of either beverage); Exercised Limb: No difference between FBP and CON; Myofibrillar protein synthesis increased within both groups with exercise (*p* < 0.05)
Pinckaers et al., 2023 ([60]) (36170964)	24 young males (aged 24 ± 4 y; mean ± SD)	30 g plant-derived protein blend combining 15 g wheat, 7.5 g corn, and 7.5 g pea protein (PLANT-BLEND)	30 g milk protein (MILK)	No exercise	Ingestion of both MILK and PLANT-BLEND increased myofibrillar protein synthesis rates (*p* < 0.001); No significant differences between treatments.
Pinckaers et al., 2024 ([14])(38228945)	24 young males (24 ± 3 y; mean ± SD)	30 g pea (PEA)	30 g milk-derived protein (MILK)	No exercise	Ingestion of both MILK and PEA increased MPS (*p* < 0.05); No significant differences between treatments.
Pinckaers et al., 2024 ([61]) (37972895)	16 older adults (8 males, 8 females) (65–85 y; mean ± SD)	Isonitrogenous and isocaloric whole-food vegan meal (PLANT)	Whole-food omnivorous meal containing beef as the primary source of protein (0.45 g protein/kg body mass; MEAT)	No exercise	Ingestion of MEAT resulted in ∼47% higher postprandial muscle protein synthesis rates when compared with the ingestion of PLANT (*p* = 0.037).
Pinckaers et al., 2024 ([62]) (38315260)	36 healthy young males (26 ± 4 y; mean ± SD)	30 g corn protein (CORN)	30 g milk protein (MILK) or a 30 g protein blend with 15 g corn plus 15 g milk protein (CORN + MILK)	No exercise	Ingestion of protein increased myofibrillar protein synthesis rates from basal post-absorptive values in all treatments (*p* < 0.05); Postprandial myofibrillar protein synthesis rates did not differ between CORN vs. MILK or CORN + MILK vs. MILK.
Monteyne et al., 2020 ([63]) (32438401)	20 resistance-trained healthy young males (age: 22 ± 1 y; mean ± SEM)	70 g (31.5 g protein/2.5 g leucine) mycoprotein (MYCO)	31 g (26.2 g protein/2.5 g leucine) milk protein (MILK)	5 × 30 maximal concentric isokinetic leg-extension and leg-flexion contractions on a Biodex System 3 isokinetic dynamometer	Mixed muscle FSRs increased following MILK ingestion (both rested and exercise) but to a greater extent following MYCO ingestion (both rested and exercise) (treatment × time interaction effect; *p* < 0.05).
Monteyne et al., 2020 ([64]) (32886108)	19 healthy males (22 ± 1 y; mean ± SEM)	70 g mycoprotein (31.5 g protein; MYCO; n = 10) OR 35 g BCAA-enriched mycoprotein (18.7 g protein: matched on BCAA content; ENR; n = 9)	N/A	5 × 30 maximal concentric isokinetic leg-extension and leg-flexion contractions on a Biodex System 3 isokinetic dynamometer	MPS increased with protein ingestion but to a greater extent following MYCO ingestion (*p* < 0.05); Postprandial MPS rates were greater in MYCO compared with ENR (*p* < 0.05).
Monteyne et al., 2021 ([65]) (33172506)	19 healthy older adults (66 ± 1 y; mean ± SEM)	3 d isoenergetic high-protein (1·8 g/kg body mass per d) diet, where the protein was provided from exclusively vegan (VEG; n 10; six males, four females; mycoprotein providing 57% of daily protein intake) sources	3 d isoenergetic high-protein (1·8 g/kg body mass per d) diet, where the protein was provided from predominantly (71%) animal (OMNI; n 9; six males, three females) sources	Unilateral leg extension exercise (5 × 30 reps of maximal concentric isokinetic knee extension contractions of their dominant leg)	Daily myofibrillar protein synthesis rates were greater in the exercised leg compared with rested leg in OMNI and VEG groups, respectively (*p* < 0.05); Daily myofibrillar protein synthesis rates did not differ between OMNI and VEG in either rested or exercised muscles.
Monteyne et al., 2023 ([66]) (36822394)	16 healthy young adults (8 males and 8 females) (23 ± 1 y; mean ± SD)	3 d dietary intervention (high protein, 1.8 g/kg body mass per d) where protein was derived from exclusively non-animal (VEG1; n = 8) sources	3 d dietary intervention (high protein, 1.8 g/kg body mass per d) where protein was derived from omnivorous (OMNI1; n = 8) sources	Unilateral leg extension exercise (5 × 30 reps of maximal concentric isokinetic knee extension contractions of their dominant leg)	Daily myofibrillar protein synthesis rates were ~12% higher in the exercised leg than in the rested leg (*p* < 0.05); Daily myofibrillar protein synthesis rates were not different between groups.
Churchward-Venne et al., 2019 ([67]) (30698812)	36 healthy young males (23 ± 0.4 y; mean ± SEM)	45 g carbohydrate + 20 g soy protein OR leucine-enriched soy (same level of leucine as in whey)	45 g carbohydrate + 20 g whey	Concurrent exercise: 4 × 8 reps @ ∼80% 1 RM + 30 min of continuous cycling at ∼60% of their previously determined maximal workload (Wmax)	Myofibrillar and mitochondrial PS rates over the entire 360 min recovery period did not differ between treatments.
West et al., 2023 ([68]) (37529834)	33 healthy, young females and males (age: 21 ± 1 y; mean ± SEM)	25 g of protein from mycoprotein (MYC, n = 11), pea protein (PEA, n = 11), or a blend (39% MYC and 61% PEA) of the two (BLEND, n = 11)	N/A	Whole-body REX program (1 × 10 reps @ 75% 10-RM + 3 × AMRAP @ 10 RM)	Resistance exercise and protein ingestion increased myofibrillar FSRs over a 4 h postprandial period (*p* < 0.05); No differences in muscle protein synthesis responses between groups.
West et al., 2023 ([69]) (36172885)	24 healthy young (age, 21 ± 2 y; mean ± SEM)	70 g mycoprotein (MYC; 31·4 g protein and 2·5 g leucine; n 12) or 38·2 g of a protein concentrate obtained from mycoprotein (PCM; 28·0 g protein and 2·5 g leucine; n 12)	N/A	5 × 30 maximal concentric isokinetic leg-extension and leg-flexion contractions on a Biodex System 3 isokinetic dynamometer	Protein ingestion increased myofibrillar FSR in both rested and exercised muscles (*p* < 0.05) with no differences between conditions; Mycoprotein ingestion results in equivalent postprandial stimulation of resting and post-exercise myofibrillar protein synthesis rates irrespective of whether being consumed within or without its whole-food matrix.
van der Heijden et al., 2023 ([70]) (37716611)	36 healthy young adults females and males (age: 22 ± 3 y; mean ± SD)	25 g protein from (fungal-derived mycoprotein [MYCO]) (n = 12), Spirulina [SPIR] (cyanobacterium) (n = 12), or chlorella [CHLO] (microalgae) (n = 12; m/f, 6/6)	N/A	4 unilateral sets of leg press, followed by 4 sets of leg extension at the predetermined 10 RM until volitional failure (8–12 repetitions)	Protein ingestion increased myofibrillar protein synthesis rates in both rested and exercised tissue (*p* < 0.05) with no differences between groups; Higher rates of muscle protein synthesis in exercised compared with rested muscle.
Lim et al., 2024 ([71]) (38846451)	8 healthy and recreationally active (men: n = 4; women: n = 4; 18–30 y; mean ± SD)	25 g WHEY plant-based blend protein (88% pea protein and 12% canola protein or plant-based blend protein + leucine matched with WHEY; PBP)	25 g WHEY (SureProtein WPI 895)	No exercise	All protein supplements increased mixed MPS above postabsorptive levels (*p* < 0.05); Magnitude of increase in MPS following ingestion of PBP was less than that following ingestion of PBP + Leu and WHEY; No differences in MPS between PBP + Leu and WHEY (*p* = 0.052).
van der Heijden et al., 2024 ([72]) (38537270)	10 resistance-trained, young adults (male/female: 8/2; age: 26 ± 6 y; mean ± SD)	32 g plant protein blend (BLEND; 39.5% pea, 39.5% brown rice, and 21.0% canola)	32 g protein from whey (WHEY)	Bilateral leg resistance exercise	Myofibrillar protein synthesis rates increased after exercise and protein ingestion over a 0 to 2 h period and 2 to 4 h period (*p* < 0.05); No differences in muscle protein synthesis between conditions during either period or throughout the entire (0–4 h) postprandial period.
Domic et al., 2024 ([73] (39732437)	34 community-dwelling older adults (18 males and 16 females) (72 ± 4 y; mean ± SD)	1.3 g/kg body mass per d protein from all vegan sources: soy-based dairy alternatives, legumes, nuts, cereals, and plant-based meat analogues based on (isolated) pea protein and/or rice protein	1.3 g/kg body mass per d protein from omnivorous sources: dairy products, cheese, chicken, beef, and pork sausage	No exercise	Integrated MPS rates did not differ between vegan (1.23 ± 0.04%/day) and omnivorous (1.29 ± 0.04%/day) diets (*p* = 0.2542).

Abbreviations: AMRAP, As Many Repetitions As Possible; RM, Repetition Maximum; MPS, Muscle Protein Synthesis; FSR, Fractional Synthetic Rate; SD, Standard Deviation; SEM, Standard Error Mean.
